# Risk factors for adverse maternal and fetal outcomes in SLE patients: a systematic review and meta-analysis

**DOI:** 10.3389/fmed.2025.1573573

**Published:** 2025-09-24

**Authors:** Hang Liu, Meifei Li, Meijiao Wang, Minzhe Ren, Jiaying Fu, Ying Cai, Zhiyu Li, Ting Zhao, Jing Sun, Zhijun Xie

**Affiliations:** ^1^College of Basic Medical Science, Zhejiang Chinese Medical University, Hangzhou, China; ^2^Department of Nephrology, the First Affiliated Hospital of Zhejiang Chinese Medical University (Zhejiang Provincial Hospital of Chinese Medicine), Hangzhou, Zhejiang, China; ^3^Zhejiang Key Laboratory of Research and Translation for Kidney Deficiency-Stasis-Turbidity Disease, Zhejiang-Macau International Joint Laboratory of Integrated Traditional Chinese and Western Medicine for Nephrology and Immunology, Hangzhou, Zhejiang, China; ^4^The Second School of Clinical Medicine, Zhejiang Chinese Medical University, Hangzhou, China; ^5^China and Zhejiang University of Traditional Chinese Medicine Jinhua Research Institute, Hangzhou, China

**Keywords:** SLE, risk factors, composite APOs, preterm birth, meta-analysis

## Abstract

**Background:**

Systemic lupus erythematosus (SLE) is a multisystem autoimmune disease that increases the risk of adverse maternal and fetal outcomes in SLE pregnancies. Identifying potential risk factors can enhance preconception risk assessment for SLE pregnancies, thereby reducing the burden of pregnancy for SLE patients.

**Objective:**

The goal of this meta-analysis is to designate the risk factors for unfavorable maternal and fetal outcomes in SLE pregnancies by means of a systematic review of the literature and meta-analysis.

**Methods:**

The odds ratios (ORs) and associated 95% confidence intervals (CIs) were estimated using either a fixed-effects model or a random-effects model. The I^2^ statistic was used to assess heterogeneity. Sensitivity analysis, Egger’s test, the Newcastle-Ottawa Quality Assessment Scale (NOS), and the Grading of Recommendations Assessment, Development, and Evaluation (GRADE) system were also performed.

**Results:**

Eleven papers with 1,790 SLE patients who were pregnant were examined in the meta-analysis out of 2,467 citations that were screened. The meta-analysis’s findings indicated that the onset of SLE is associated with an increased risk of preterm birth (OR: 2.85; 95% CI: 2.04, 3.99). Hypertension is associated with an increased risk of composite pregnancy outcomes (OR: 4.56; 95% CI: 2.42, 8.53), preterm birth (OR: 2.20; 95% CI: 1.53, 3.17) and preeclampsia (OR: 10.11; 95% CI: 1.83, 55.89). Renal involvement is associated with an increased risk of composite pregnancy outcomes (OR: 3.09; 95% CI: 1.66, 5.72) and preterm birth (OR: 1.65; 95% CI: 1.22, 2.23). Anti-dsDNA is associated with an increased risk of preterm birth (OR: 1.83; 95% CI: 1.13, 2.92) and pregnancy loss (OR: 2.64; 95% CI: 1.09, 6.40). Drug therapy is associated with a decreased risk of composite pregnancy outcomes (OR: 0.51; 95% CI: 0.31, 0.85), preterm birth (OR: 0.66; 95% CI: 0.48, 0.89) and pregnancy loss (OR: 0.42; 95% CI: 0.21, 0.84). Sensitivity analysis demonstrated how solid our results are. Egger’s test revealed no discernible publication bias.

**Conclusion:**

The onset of SLE, hypertension, renal involvement, drug therapy, and serological factors have a predictive effect on the occurrence of adverse maternal and fetal outcomes in SLE pregnancies. Strengthening preconception risk assessment for SLE patients plays an important role in reducing pregnancy risks and improving the quality of life during pregnancy.

**Systematic review registration:**

https://www.crd.york.ac.uk/prospero/#recordDetails, identifier: CRD42024564190.

## 1 Introduction

Systemic lupus erythematosus (SLE) is a multisystem, connective tissue autoimmune illness that affects female patients who are of reproductive age and has a negative impact on both the mother and the fetus (1). Research indicates that SLE significantly raises the likelihood of adverse pregnancy outcomes (APOs), and that pregnancy may cause the onset or progression of SLE (2). Compared to ordinary pregnant women, pregnant women with SLE are more prone to APOs, characterized by maternal complications (lupus flares, kidney damage, preeclampsia (PE), etc.) and fetal complications (preterm birth (PTB), pregnancy loss (PL), intrauterine growth retardation (IUGR), small for gestational age (SGA), low birth weight (LBW), and neonatal lupus (NL), etc.) (3). Due to advancements in preconception and prenatal treatment, SLE patients now have better pregnancy outcomes. Nevertheless, pregnant SLE patients frequently have dismal results (4). Some measures can be taken, such as using assisted reproductive technologies and monitoring disease activity (5), to lower the risk of poor outcomes in people with SLE. Nonetheless, both doctors and patients continue to find it difficult and complex to diagnose and treat pregnant SLE patients (6). Thus, for patients with SLE pregnancies, proactive and efficient risk factor identification and management are crucial.

Some published meta-analysis have been done on related subjects. Sepsis, hypertension, lupus nephritis (LN), PE, induced abortion, and PTB are common in patients with SLE pregnancies, according to a meta-analysis involving 1,842 patients. Additionally, patients with LN or positive antiphospholipid antibody (aPL) are more likely to experience PTB (7). Patients with SLE pregnancies had an elevated risk of PE, hypertension, spontaneous abortion (SA), and postpartum infections, according to another meta-analysis involving 529,788 patients (8). However, the relationship between risk factors and these unfavorable outcomes has not been comprehensively investigated. In this meta-analysis, we focused on pregnant patients with SLE from different countries and races, with a particular emphasis on the effects of new or recurrent SLE, renal involvement, hypertension, drug therapy, Systemic Lupus Erythematosus Disease Activity Index (SLEDAI), and serological factors on APOs, PTB, PE, and PL. It helps to conduct preconception risk assessment and testing for pregnant patients with SLE, thereby improving adverse maternal and fetal outcomes and enhancing quality of life during pregnancy. At the same time, the Grading of Recommendations Assessment, Development, and Evaluation (GRADE) system was used to evaluate the overall certainty of the evidence, which helps clinical doctors interpret the research results and also assists researchers in identifying the lack of evidence, clarifying the direction and priority of future research (9). We also discussed potential mechanisms and made recommendations for future research.

## 2 Methods

The Preferred Reporting Items for Systematic Reviews and Meta-Analysis (PRISMA) 2020 guidelines (10) were followed in the reporting of this systematic review and meta-analysis, and our protocol was registered on PRPSPERO on July 13, 2024 (CRD42024564190).

### 2.1 Data sources

PubMed, EMBASE, Web of Science and Cochrane Library were searched from 1988 to July 2024 with no restrictions on location. We searched using relevant keywords and subject terms. SLE, risk factors, pregnancy, and their variations were among the search phrases used. Supplementary Table S1 contains the entire search strategy for these databases.

### 2.2 Study selection

Original records were loaded into EndNote, and duplicate records were eliminated. Overall, titles and abstracts were separately examined by different authors. To ascertain whether an article satisfied the inclusion requirements, the entire text was examined. Disagreements would be resolved through discussion among all authors.

### 2.3 Eligibility criteria

Studies were approved provided they fulfill these requirements: (a) patients: adult pregnant patients (≥18 years) with SLE; (b) exposure: a conclusive diagnosis of SLE; (c) experimental group: pregnant SLE patients with unfavorable outcomes; control group: pregnant SLE patients with no unfavorable outcomes; (d) outcomes: the 95% confidence intervals (CIs) for either adjusted or unadjusted odds ratios (ORs); and (e) study type: cohort study and case–control study. These were the conditions for exclusion: (a) duplicate publications; (b) conference abstracts; (c) review articles; (d) animal experimental studies; (e) case reports; (f) letters; (g) incomplete data; (h) no full-text article, (i) no results of interest and (j) no appropriate experimental group or control group.

### 2.4 Data extraction

We used Microsoft Excel (Microsoft Corporation, United States) to create a data extraction form. In total, two authors (Hang Liu and MF Li) worked together to extract data from all the relevant studies separately. The data below were extracted from every study: first author, publication year, country, study type, sample size, follow-up time, mean age at conception, adverse outcomes, risk factors, and OR with 95% CI. Disagreements were settled by reaching a consensus.

### 2.5 Study quality

Three dimensions of quality were assessed for each of the studies using the NOS: selection, comparability, and outcome (11). Cohort and case–control study ratings ranged from 0 to 9 stars, where higher ratings denoted higher quality research (12). The quality of the included studies was classified as high, moderate, and low (12).

### 2.6 Evidence certainty

The GRADE approach (13) was proposed as a way to assess the total evidence certainty. The certainty of the evidence obtained from queue studies was initially rated as low quality according to this system (9). When the impact of cohort studies is significant enough, or when influenced by dose–response gradients and reasonable confounding factors, the evidence quality will also be enhanced following the elimination of several elements that could result in a downgrade (14). Finally, the evidence for the results were classified as high, medium, low, or very low (14).

### 2.7 Statistical analysis

Statistical analyses were done using Stata software (version 14). We employ the chi-square test and I^2^ value to evaluate heterogeneity. *p* < 0.05 or I^2^ > 50% indicates strong heterogeneity, and random effects should be chosen (15). Otherwise, choose a fixed-effects model. Perform a sensitivity analysis to confirm the overall results’ resilience and investigate the causes of heterogeneity. Finally, to identify publication bias, Egger’s test and funnel plots were employed (16).

## 3 Results

### 3.1 Study selection

**Figure 1:**
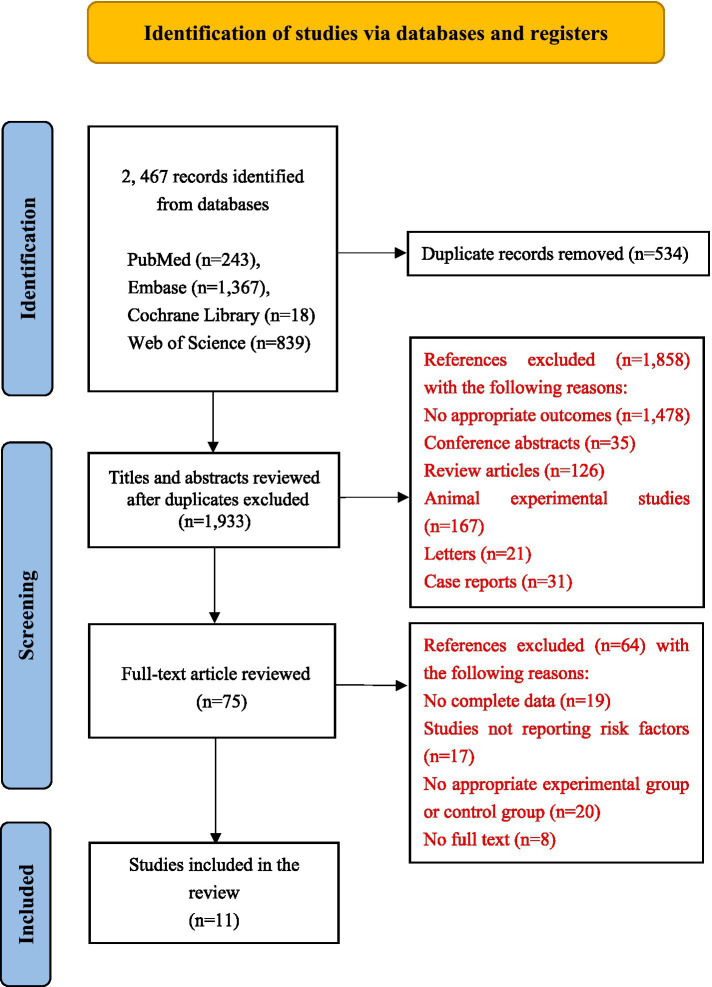
Flow chart of the process of study selection of PRISMA.

Our study found 2,467 related articles, of which 534 were removed as duplicates. Of these, 1,858 were checked for titles and abstracts unrelated to our topic. The final 75 articles were carefully examined for additional evaluation. Finally, 10 retrospective cohort studies (1, 6, 17–24) and one retrospective case–control study (4) were included in the meta-analysis. The process of study selection is described in Figure 1.

### 3.2 Study characteristics

The studies that are considered were conducted on a total of 1,790 patients with SLE pregnancies between 1988 and 2024. Retrospective cohort design (1, 6, 17–24) was employed in 10 studies, and retrospective case–control design (4) was utilized in one study. Studies were undertaken in China (*n* = 5) (1, 6, 18–20), the United States (*n* = 2) (17, 22), Thai (*n* = 1) (20), Japan (*n* = 1) (23), Indonesia (*n* = 1) (4), and Malaysia (*n* = 1) (24). Seven studies (4, 17, 19–23) used the 1997 Revised American College of Rheumatology (ACR) criteria (25) for SLE. Three studies (1, 6, 24) used the 1997 ACR classification criteria or the 2012 Systemic Lupus International Collaborating Clinics classification criteria (25, 26) for SLE, and one study (18) used the 2009 ACR classification criteria (25) for SLE. The general details of the studies are shown in Table 1.

**Table 1:** General information of the studies included in this meta-analysis.

Reference	Publication year	Country	Study type	Sample Size	Follow-up period	Mean age at conception (years)	Adverse outcomes	Risk factors	Effect size
Jian Chen et al.	2020	China	Retrospective cohort study	Pregnant women: 85 Pregnancies: NA	2011–2018	27.4 (16,41)	PTB, Composite APOs, PE, PL, FGR	PTB: (1), (2), (3), (4), (5), (6), (8), (29) Composite APOs: (1), (2), (3), (4), (5), (6), (8), (29) PE: (1), (2), (3), (4), (5), (6), (8), (29) PL: (1), (2), (3), (4), (5), (6), (8), (29) FGR: (1), (2), (3), (4), (5), (6), (8), (29)	OR (95% CI)
Eliza F. Chakravarty et al.	2005	United States	Retrospective cohort study	Pregnant women: 48 Pregnancies: 63	1991–2001	30 ± 6.5	PE, PTB	PE: (1), (2), (4), (7), (9), (10), (11), (14) PTB: (1), (2), (4), (7), (9), (10), (11), (14)	OR (95% CI)
Dongying Chen et al.	2020	China	Retrospective cohort study	Pregnant women: 243 Pregnancies: NA	2011–2016	28.9 ± 3.9	PTB, IUGR, Composite APOs	PTB: (3), (30) IUGR: (3) Composite APOs: (7)	OR (95% CI)
Meng Jiang et al.	2021	China	Retrospective cohort study	Pregnant women: 484 Pregnancies: 513	2010–2018	29.8 ± 4	PTB, SGA, BA	PTB: (2), (3), (30) SGA: (3), (16), (30) BA: (3)	OR (95% CI)
Worawit Louthrenoo et al.	2021	Thai	Retrospective cohort study	Pregnant women: 77 Pregnancies: 90	1993–2017	26.94 ± 4.80	PTB, PL, SGA, LBW	PTB: (1), (2), (3), (12), (17), (18) PL: (1), (2), (3), (12), (17), (18) SGA: (1), (2), (3), (12), (17), (18) LBW: (1), (2), (3), (12), (17), (18)	OR (95% CI)
Kensuke Irino et al.	2021	Japan	Retrospective cohort study	Pregnant women: 39 Pregnancies: 64	2009–2016	31.2 ± 4.9	PTB	PTB: (2), (7), (9), (11), (12), (14), (19)	OR (95% CI)
Ke Zhang et al.	2021	China	Retrospective cohort study	Pregnant women: 123 Pregnancies: 123	2014–2020	27.1 ± 4.1	Composite APOs, PL, PTB, LBW	Composite APOs: (2), (14), (20) PL: (2), (14) PTB: (14), (20) LBW: (20)	OR (95% CI)
Laniyati Hamijoyo et al.	2019	Indonesia	Retrospective case–control study	Pregnant women: 84 Pregnancies: 109 (case: 54; control: 55)	2016–2018	28 ± 3	Composite APOs	Composite APOs: (1), (3), (10), (21)	OR (95% CI)
Qianwen Dai et al.	2024	China	Retrospective cohort study	Pregnant women: 408 Pregnancies: 445	2010–2023	31 (28, 33)	PTB, Composite APOs, PE	PTB: (2), (3), (30) Composite APOs: (3) PE: (3), (13), (20), (22), (23)	OR (95% CI)
Syahrul S Shaharir et al.	2020	Malaysia	Retrospective cohort study	pregnant women: 153 Pregnancies: 240	2016–2019	29.9 ± 4.8	PTB, Composite APOs, PE, IUGR	PTB: (1), (2), (20), (24), (25) Composite APOs: (1), (2), (3), (24), (25), (26) PE: (1), (2), (3), (9), (20), (24) IUGR: (20), (24), (29)	OR (95% CI)
Masashi Deguchi et al.	2018	United States	Retrospective cohort study	pregnant women: 46 Pregnancies: 56	2009–2016	33.9 ± 4.6	PTB, PL	PTB: (1), (4), (7), (14), (15), (27), (28) PL: (1), (4), (7), (14), (15), (27),	OR (95% CI)

(1) Renal involvement; (2) Drug therapy; (3) Hypertension; (4) SLEDAI; (5) Diagnostic delay; (6) APO history; (7) APL; (8) Albumin; (9) Active disease at conception; (10) Thrombocytopenia; (11) Anti-SSA; (12) Disease duration prior to conception; (13) APS; (14) Anti-dsDNA; (15) Disease duration of SLE; (16) Multiple pregnancy; (17) Previous pregnancy; (18) Organ involvement during Pregnancy; (19) Maternal concomitant disease; (20) PE; (21) Neuropsychiatric SLE; (22) Regular follow-up; (23) 24-h proteinuria; (24) Active hematology in pregnancy; (25) Active disease pre-pregnancy; (26) Duration of remission; (27) Laboratory findings; (28) Hypocomplementaemia-C3; (29) ACL; (30) Disease flare-up.

### 3.3 Methodological quality of studies

The 10 retrospective cohort studies (1, 6, 17–24) and one retrospective case–control study (4) that scored ≥7 demonstrate the high quality of the research, and details of the NOS are described in Table 2.

**Table 2:** Newcastle-Ottawa quality of studies.

Study type	Authors	Selection	Comparability	Outcome	Overall quality score	Classification
Cohort study	Chen et al. (19)	***	**	***	********	High quality
	Chakravarty et al. (17)	***	**	***	********	High quality
	Chen et al. (19)	***	**	**	*******	High quality
	Jiang et al. (20)	***	**	***	********	High quality
	Louthrenoo et al. (21)	***	**	***	********	High quality
	Irino et al. (23)	***	**	***	********	High quality
	Zhang et al. (36)	***	**	***	********	High quality
	Dai et al. (1)	***	**	***	********	High quality
	Shaharir et al. (24)	***	**	**	*******	High quality
	Deguchi et al. (22)	***	**	***	********	High quality
Case–control study	Hamijoyo et al. (4)	***	**	***	********	High quality

### 3.4 Definitions of adverse maternal and fetal outcomes

Composite APOs have no specific definition, and it was defined as any number ≥2 of unfavorable pregnancy outcomes. PTB is a common fetal outcome, defined as delivery before 37 weeks of gestation (22, 24, 27). Following 20 weeks of pregnancy, PE is defined as newly established hypertension (systolic blood pressure >140 mmHg or diastolic blood pressure >90 mmHg) combined with or without proteinuria (0.3 g/24 h) (28). Different studies define PE differently. In addition to elevated blood pressure and proteinuria, with or without other organ dysfunction, including kidney, liver, and placental dysfunction, it is also included in the definition of PE (1). The definition of PL varies slightly in different studies, but overall, PL encompasses stillbirth, infant mortality, therapeutic, spontaneous, and selective abortion (22, 24).

### 3.5 Interpretations of factors

All factors included were described in Table 3.

**Table 3:** Definitions of factors included in this meta-analysis.

Factors	Definitions
Renal involvement	LN or a history of LN is defined as renal involvement. Clinical and laboratory signs of LN are those that satisfy the criteria set forth by the ACR (sustained proteinuria higher than 0.5 g per day, test strip values greater than 3+, and/or cell cast, including hemoglobin, granular, tubular, or mixed red blood cells) (55).
Drug therapy	Drug therapy in this meta-analysis includes aspirin and hydroxychloroquine (HCQ).
Hypertension	In this study, hypertension refers to both gestational hypertension (GH) and pre-existing hypertension. Systolic blood pressure ≥140 mmHg and/or diastolic blood pressure ≥90 mmHg were considered pre-existing hypertension. After 20 weeks of gestation, new-onset blood pressure ≥140/90 mmHg without proteinuria is known as GH (56).
SLEDAI grade	There are four groups of SLEDAI scores: SLEDAI scores of 5–9 for mild activity, 10–14 for moderate activity, and 15 or higher for severe activity (46).
Anti-dsDNA	Anti-dsDNA antibody has been a part of the classic diagnostic and nosological criteria for SLE since 1982, and it is thought to be a specific antibody for SLE (57).
Anti-SSA	Anti-SSA, the characteristic antibody of Sjögren’s syndrome (SS), is detected in 24–60% of SLE patients and is linked to neonatal SLE (52).
Disease flare-up	Disease flare-up was defined in accordance with the International Consensus for disease flare-up in SLE, which included new onset or progression of SLE (58).

### 3.6 Results of synthesis

The results of the association between risk factors and composite APOs, PTB, PE and PL are described separately.

#### 3.6.1 Composite APOs

**Figure 2:**
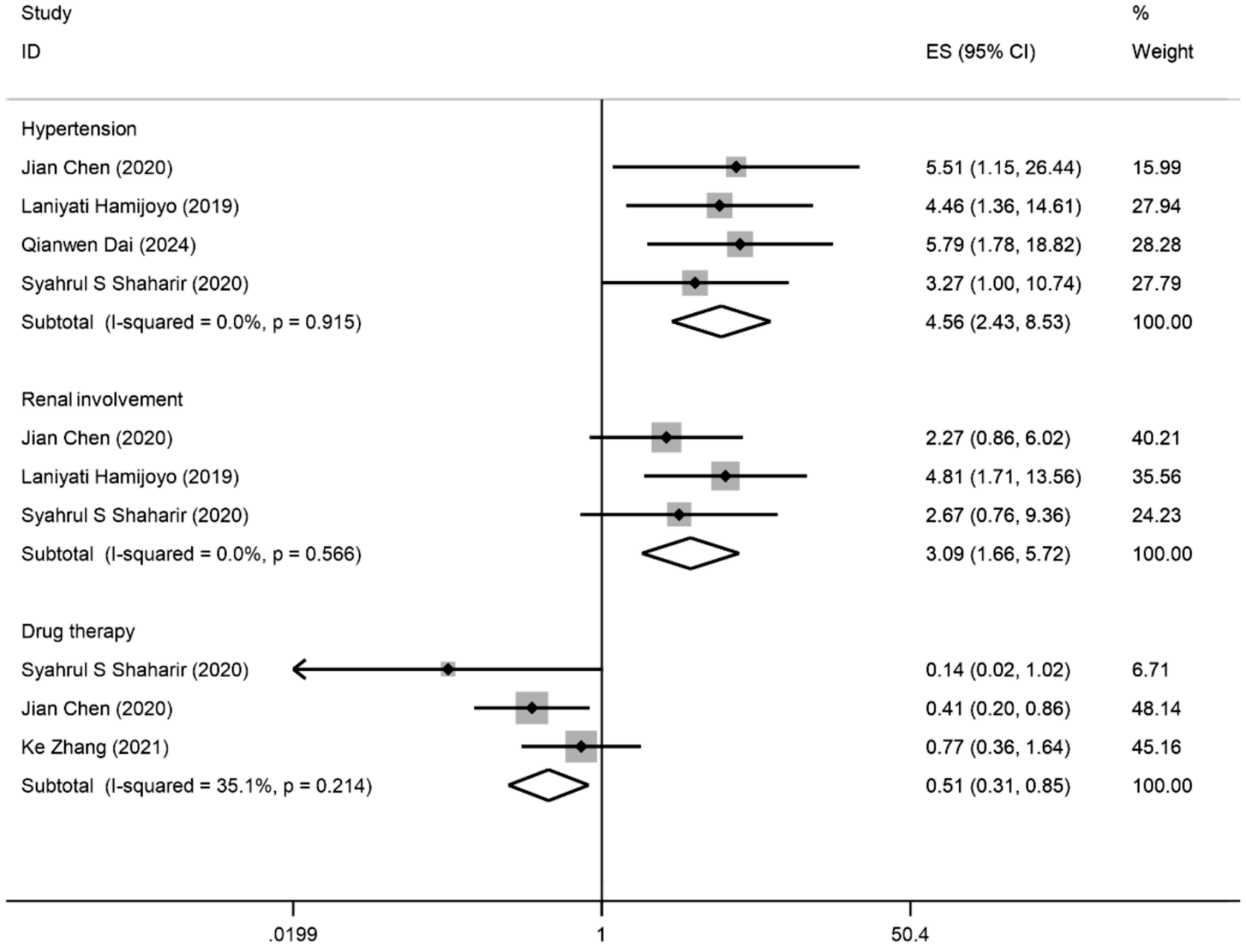
Forest plot presents the association between factors and composite APOs: odds ratios (ORs) with 95% confidence intervals (CIs).

Five studies (1, 4, 6, 18, 24) involving a total of 1,096 patients reported three predictors of composite APOs. Of these five studies, four studies (1, 4, 18, 24) indicated hypertension as a predictor, three studies (4, 18, 24) indicated renal involvement as a predictor, and three studies (6, 18, 24) indicated drug therapy as a predictor of composite APOs. The findings of the meta-analysis (Figure 2) demonstrated that hypertension (OR: 4.56; 95% CI: 2.42, 8.53; I^2^ = 0.0%; *p* = 0.000) and renal involvement (OR: 3.09; 95% CI: 1.66, 5.72; I^2^ = 0.0%; p = 0.000) were linked to an elevated risk of composite APOs. Drug therapy (OR: 0.51; 95% CI: 0.31, 0.85; I^2^ = 35.1%; *p* = 0.009) was related to a reduced risk of composite APOs. In each study, we conducted a sensitivity analysis, and the results were robust after excluding any of the studies, so our results were stable. The visual inspection funnel plot was relatively symmetric, indicating little sign of publication bias (Supplementary Figure S1). Furthermore, Egger’s test (*p* = 0.216) revealed no discernible publication bias.

#### 3.6.2 Preterm birth

Ten studies (1, 6, 17–24) involving a total of 1,706 patients reported six predictors of PTB. Of these studies, four studies (1, 19, 20, 23) indicated disease flare-up as a predictor, five studies (17, 18, 21, 22, 24) showed renal involvement as a predictor, five studies (1, 17, 20, 21, 24) showed drug therapy as a predictor, five studies (1, 18–21) showed hypertension as a predictor, three studies (6, 17, 22) showed that anti-dsDNA was a predictor, and two studies (17, 18) showed that anti-SSA was a predictor of PTB. The findings of the meta-analysis (Supplementary Figure S2) demonstrated that disease flare-up (OR: 2.85; 95% CI: 2.04, 3.99; I^2^ = 8.3%; *p* = 0.000), renal involvement (OR: 1.65; 95% CI: 1.22, 2.23; I^2^ = 41.4%; *p* = 0.001), hypertension (OR: 2.20; 95% CI: 1.53, 3.17; I^2^ = 47.8%; *p* = 0.000), and anti-dsDNA (OR: 1.83; 95% CI: 1.13, 2.92; I^2^ = 28.6%; *p* = 0.014) were linked to an increased risk of PTB. Drug therapy (OR: 0.66; 95% CI: 0.48, 0.89; I^2^ = 42.5%; *p* = 0.006) was related to a reduced risk of PTB. Anti-SSA (OR: 0.78; 95% CI: 0.51, 1.18; I^2^ = 0.0%; *p* = 0.241) was not a statistically significant factor. In each study, we conducted a sensitivity analysis, and when we excluded any studies, the results were robust, indicating that the results were stable. The funnel plot (Supplementary Figure S3) gave out a relatively symmetrical pattern, indicating little sign of publication bias. In addition, Egger’s test (*p* = 0.422) revealed no substantial publication bias.

#### 3.6.3 Preeclampsia

Four studies (1, 17, 18, 24) with a total of 694 patients reported five predictors of PE. Three studies showed drug therapy (17, 18, 24) and renal involvement (17, 18, 24) as predictors, three studies (1, 18, 24) showed hypertension as a predictor, and two studies (17, 18) showed SLEDAI and anti-SSA as predictors of PE. The findings of the meta-analysis (Supplementary Figure S4) demonstrated that hypertension (OR: 10.11; 95% CI: 1.83, 55.89; I^2^ = 83.6%; *p* = 0.008) was linked to the progression of PE. Drug therapy (OR: 0.73; 95% CI: 0.38, 1.41, I^2^ = 17.3%; *p* = 0.349), renal involvement (OR: 2.44; 95% CI: 0.94, 6.35; I^2^ = 39.7%; *p* = 0.067), SLEDAI (OR: 1.44; 95% CI: 0.75, 2.77; I^2^ = 74.9%; *p* = 0.268) and anti-SSA (OR: 0.87; 95% CI: 0.42, 1.81; I^2^ = 0.0%; *p* = 0.708) were not statistically significant factor. In each study, we conducted a sensitivity analysis, and when we excluded any studies, the results were robust, indicating that the results were stable. The visual inspection funnel plot (Supplementary Figure S5) was relatively symmetrical, indicating little sign of publication bias. In addition, Egger’s test (*p* = 0.097) revealed no substantial publication bias.

#### 3.6.4 Pregnancy loss

Four studies (6, 18, 21, 22) with a total of 331 patients reported five predictors of PL. Three studies (18, 21, 22) showed that renal involvement was a predictor, three studies (6, 18, 21) showed that drug therapy was a predictor, two studies (6, 22) showed that anti-dsDNA was a predictor, two studies (18, 21) showed hypertension as a predictor, and two studies (18, 22) showed SLEDAI as a predictor of PL. The findings of the meta-analysis (Supplementary Figure S6) demonstrated that anti-dsDNA (OR: 2.64; 95% CI: 1.09, 6.40; I^2^ = 0.0%; *p* = 0.031) was linked to the progression of PL. Drug therapy (OR: 0.42; 95% CI: 0.21, 0.84; I^2^ = 0.0%; *p* = 0.015) was associated with a reduced risk of PL. Renal involvement (OR: 2.43; 95% CI: 0.71, 8.36; I^2^ = 44.6%; *p* = 0.160), SLEDAI (OR: 1.67; 95% CI: 0.95, 2.95; I^2^ = 0.0%; *p* = 0.077), and hypertension (OR: 0.94; 95% CI: 0.26, 3.33; I^2^ = 59.5%; *p* = 0.922) were risk factors that were not statistically significant. In each study, we conducted a sensitivity analysis, and when we excluded any studies, the results were robust, indicating that the results were stable. The visual inspection funnel plot (Supplementary Figure S7) was a relatively symmetrical pattern, indicating little sign of publication bias. In addition, Egger’s test (*p* = 0.700) revealed no substantial publication bias.

### 3.7 Evidence certainty

The evidence level is low for the risk of SLE with hypertension, SLE with renal involvement, and SLE with drug therapy in composite APOs. The evidence level is low for the risk of SLE with renal involvement, SLE with hypertension, SLE with disease flare-up, SLE with drug therapy, SLE with anti-dsDNA, and SLE with anti-SSA in PTB. The evidence level is very low for the risk of SLE with SLEDAI and SLE with hypertension, and is low for the risk of SLE with renal involvement, SLE with drug therapy, and SLE with anti-SSA in PE. The evidence level is very low for the risk of SLE with hypertension, and is low for the risk of SLE with drug therapy, SLE with renal involvement, SLE with anti-dsDNA, and SLE with SLEDAI in PL. GRADE evidence certainty for risk factors for unfavorable outcomes is summarized in Table 4.

**Table 4:** Grade certainty of evidence.

Outcome	Exposure	Study members	Grade					Evidence quality
			Risk of bias	Inconsistency	Indirectness	Imprecision	Publication bias	
PTB	SLE with disease flare-up	4	0	0	0	0	0	Low
PTB	SLE with renal involvement	3	0	0	0	0	0	Low
PTB	SLE with drug therapy	5	0	0	0	0	0	Low
PTB	SLE with hypertension	5	0	0	0	0	0	Low
PTB	SLE with anti-dsDNA	3	0	0	0	0	0	Low
PTB	SLE with anti-SSA	2	0	0	0	0	0	Low
Composite APOs	SLE with hypertension	4	0	0	0	0	0	Low
Composite APOs	SLE with renal involvement	3	0	0	0	0	0	Low
Composite APOs	SLE with drug therapy	3	0	0	0	0	0	Low
PE	SLE with drug therapy	3	0	0	0	0	0	Low
PE	SLE with renal involvement	3	0	0	0	0	0	Low
PE	SLE with hypertension	3	0	−1^a^	0	0	0	Very low
PE	SLE with SLEDAI	2	0	−1^a^	0	0	0	Very low
PE	SLE with anti-SSA	2	0	0	0	0	0	Low
PL	SLE with drug therapy	3	0	0	0	0	0	Low
PL	SLE with renal involvement	3	0	0	0	0	0	Low
PL	SLE with anti-dsDNA	2	0	0	0	0	0	Low
PL	SLE with SLEDAI	2	0	0	0	0	0	Low
PL	SLE with hypertension	2	0	−1^a^	0	0	0	Very low

^a^High heterogeneity.

## 4 Discussion

### 4.1 Main findings

In this meta-analysis, 11 studies totaling 1,790 SLE patients were considered, which examined the relationships between risk factors and composite APOs, PTB, PE, PL for mothers and fetuses. In patients with SLE pregnancies, a higher incidence of PTB was linked to SLE recurrence or new-onset SLE. An elevated risk of composite APOs, PTB, and PE was linked to hypertension. An elevated risk of composite APOs and PTB was linked to renal involvement during pregnancy or a history of the condition. Anti-SSA was not substantially correlated with PE or PTB. Anti-dsDNA was linked to an elevated risk of PTB and PL. SLEDAI was not substantially correlated with PE or PL. Drug therapy, such as aspirin and hydroxychloroquine (HCQ), has been linked to a lower incidence of composite APOs, PTB, and PL. Our meta-analysis revealed some knowledge of the risk factors that could lead to unfavorable outcomes for both mothers and fetuses: disease flare-up is a risk factor for PTB; hypertension is a risk factor for composite APOs, PTB, and PE; renal involvement is a risk factor for composite APOs and PTB; anti-dsDNA is a risk factor for PTB and PL; and drug therapy is a protective factor for composite APOs, PTB, and PL. More research is required to discover extra common and potential risk factors for unfavorable outcomes in patients with SLE pregnancies.

### 4.2 Interpretation of findings

The new onset or worsening of SLE during pregnancy is the main risk factor for PTB. According to a review (7), the incidence of PTB in female patients with SLE is much higher than that in women of good health. There are several ways in which SLE can induce spontaneous PTB. For example, activation of the hypothalamic–pituitary axis in the mother or fetus can lead to an increase in placental corticotropin-releasing hormone, which in turn promotes delivery by producing prostaglandins and cortisol (29). Emotional or physiological stress in SLE patients, or vascular dysfunction in the placenta, can also lead to PTB (30). PTB may also be associated with an unexpected decrease in estrogen levels during mid-pregnancy (31). The placenta produces a large amount of estrogen throughout pregnancy, and estrogen levels below normal may be a sign of placental dysplasia (30). Inflammation caused by local or complete infection can also induce childbirth through activation of cytokines, prostaglandins, and complement (32).

The mechanism by which SLE pregnant women with hypertension experience adverse pregnancy outcomes is mainly due to lupus-related immune dysregulation and chronic inflammation exacerbating endothelial damage (33), combined with hypertension-induced vasospasm and placental ischemia, leading to placental dysfunction, thrombosis, and fetal hypoxia, thereby significantly increasing the risk of PTB and PL (34). When placental ischemia occurs, many bioactive substances and inflammatory cytokines targeting endothelial cells are released. Subsequently, systemic endothelial cell failure occurs, leading to increased arterial stiffness, vascular remodeling, and hypertension, which in turn triggers PE (21). Studies have shown that women with hypertension have a higher risk of developing PTB and PL than those without hypertension, and high diastolic blood pressure levels are a major factor in the development of PE (35, 36). Although hypertension may theoretically increase the risk of PL, the meta-analysis did not find a significant association, which may be related to the different definitions of hypertension and PL included in the study.

Pregnant women with LN may experience deposition of immune complexes and activation of complement, damaging the endothelium of placental blood vessels, and inadequate remodeling of the uterine spiral artery, leading to reduced placental perfusion and fetal hypoxia, PL and PTB (3). Endothelial dysfunction leads to vasoconstriction and hypertension, and promotes PE by creating an imbalance between endothelium-derived vasoconstrictors and vasodilators (33). SLE, LN, diabetes and other diseases that are prone to lead to endothelial dysfunction will increase the risk of PE (30). Research has shown that women with LN have a higher risk of developing PL compared to those without LN (7). According to a meta-analysis involving 2,751 pregnant SLE patients, in 70% of cases, renal activity and proteinuria during pregnancy were predictive factors for PTB, with a 15% increase in proteinuria increasing the likelihood of PTB by 15% (37, 38). Research has shown that the incidence of PE in women with nephritis may be twice that of healthy individuals (39). This study did not find a significant association between renal involvement and PE and PL, possibly due to the fact that the manifestations of PE and LN are very similar, making it difficult to distinguish between these two processes, which can be distinguished by PIGF and sFlt-1 levels (30), but most studies have not indicated whether these biomarkers are used. The definition of renal involvement varies in different studies, and non-active LN may lower the association.

HCQ is an autophagy inhibitor that has multiple benefits in reducing the onset and occurrence of adverse outcomes in SLE (40). During the onset of SLE, the increase of VLDL-P, LDL, and triglyceride (TG) and the decrease of HDL-P levels further lead to atherosclerosis and accelerate the development of adverse outcomes (41). HCQ has thromboprotective properties that can reduce LDL and TG levels, increase HDL levels, alleviate SLE attacks, and slow down adverse outcomes (41). Studies have shown that HCQ can be safely used in pregnant SLE patients and reduce lupus activity, and the incidence rate of PL, LBW or PTB does not increase significantly (42, 43). Aspirin is a widely used NSAID that can promote placental production in early pregnancy, improve placental circulation, and potentially inhibit placental invasion of the uterine wall by altering the ratio of prostacyclin to thromboxane, thereby improving PL (3). Aspirin can also inhibit abnormal activation of complement and serum thromboxane B2, reverse platelet-induced coagulation cascade reactions, and improve endothelial dysfunction (44). Research has shown that when aspirin is taken before 16 weeks of pregnancy, the incidence of PTB and PE decreases by 90% (45). Another study also suggests that aspirin treatment is a protective factor for PL and PTB (21), which is consistent with our research findings.

SLEDAI is the most common scale for evaluating SLE activity (46). Lupus activity significantly increases the risk of APOs, mainly through immune dysfunction (47), endothelial damage (34), and placental dysfunction (33). When active lupus is combined with antiphospholipid antibody syndrome (APS), immune complex deposition and complement activation trigger vascular inflammation, leading to endothelial dysfunction, vasoconstriction, and placental ischemia, promoting the occurrence of PE (34). At the same time, antiphospholipid antibodies (aPLs) promote thrombus formation and inhibit trophoblast invasion, causing placental hypoperfusion and increasing the risk of PTB and PL (34). Research shows that the baseline SLEDAI score in pregnancy is positively correlated with the risk of PTB, and every unit of increase in SLE score will increase the incidence rate of PTB by 60% (48). Among women with moderate to severe lupus activity, pregnant women with active lupus have a higher risk of developing PE and PL (49). The meta-analysis did not find a significant association between SLEDAI and PE and PL, which may be due to insufficient sensitivity of SLEDAI to pregnancy-specific pathology or insufficient correction for confounding factors such as aPL. However, the biological association cannot be ignored.

Anti-dsDNA antibody is specific biomarkers for diagnosing SLE and are closely related to the occurrence of LN (3). Anti-dsDNA antibody forms visible immune complexes in the glomerulus, which activates complement, leading to infiltration of inflammatory cells and tissue loss, promoting the development of LN (30). Elevated anti-dsDNA antibody, especially when combined with active lupus, increases the risk of PTB (50). The study indicated that positivity of anti-dsDNA antibody in SLE pregnant women is a risk predictor for PL and PTB (51). Anti-SSA is an essential anti-nuclear and predictive antibody for adverse outcomes in SLE patients (3). Research has shown that neonatal lupus syndrome (NTE) is a disease associated with anti-SSA antibody that can damage the fetal heart during pregnancy, leading to abnormalities in the skin, liver, and blood system (52). A study on lupus mortality and anti-SSA cardiac manifestations in 18 newborns showed that 17 fetuses died, mostly in late pregnancy (53). The results of this meta-analysis showed that anti-SSA is not significantly associated with PTB and PE, and there is no research that can confirm the direct association between anti-SSA and PE and PTB. However, when anti-SSA antibody is combined with active lupus, they indirectly increase the risk of APOs in SLE patients (54).

### 4.3 Strengths and limitations

This is the first meta-analysis to comprehensively analyze the correlation between risk factors and unfavorable maternal and fetal outcomes in SLE pregnancies. The idea that risk factors predict unfavorable outcomes in SLE patients, as well as ways to lessen the burden on patients with SLE pregnancies, has garnered increasing attention, as seen by the nine studies that have been published in the last 5 years.

This meta-analysis has certain limitations. Firstly, in our meta-analysis, there is a certain degree of heterogeneity in the larger I^2^. Although sensitivity analysis indicates that the results are relatively stable, this inevitably affects the credibility of the results and also leads to low evidence quality. The reason for heterogeneity may be due to differences in SLE diagnosis (1997 ACR classification criteria, 2012 Systemic Lupus International Collaborating Clinics classification criteria, or 2009 ACR classification criteria), patient inclusion criteria (different countries, races, ages, and number of pregnancies), risk factors (history or onset of LN, new or recurrent SLE, gestational hypertension or pre-existing hypertension, and use of HCQ or aspirin), and definitions of maternal and fetal outcomes (such as composite APOs, PE, and PL). Composite APOs have no specific definition, and it was defined as any number ≥2 of unfavorable pregnancy outcomes. In addition to the conventional symptoms of elevated blood pressure and proteinuria, some studies also include organ damage in the definition of PE (1, 28). PL varies slightly in different studies, but overall, it includes still birth, infant mortality, therapeutic, spontaneous, and selective abortion (22, 24). Moreover, some of the included studies did not offer corrected 95% CIs. We reached out to the respective authors of the original studies to get details on the missing data, but none of them responded, which to some extent affected the credibility of the results. Thirdly, retrospective studies are susceptible to residual confounding predictors, and small sample studies may also have unstable results due to random errors, which reduces the association between risk factors and adverse maternal and fetal outcomes. In addition, the limited data in the original study were insufficient to analyze demographic factors (age, economic income, education level, occupation, etc.), other clinical risk factors (APS, etc.), and serological factors (anti-Smith antibodies, low C3, and low C4, etc.). In the future, more rigorously designed and sufficiently sampled prospective cohort studies are needed to further validate the association between risk factors and maternal and fetal outcomes in SLE patients.

## 5 Conclusion

To improve the prognosis of SLE pregnancies, special emphasis should be given to identifying and controlling risk factors for unfavorable pregnancy outcomes. Our findings highlight seven risk factors associated with adverse outcomes in SLE pregnancies. Further studies are needed to clarify the role of additional demographic, clinical and serologic predictors.

## Data Availability

The original contributions presented in the study are included in the article/Supplementary material, further inquiries can be directed to the corresponding authors.
