# Supplementary material for: Risk factors for adverse maternal and fetal outcomes in SLE patients: a systematic review and meta-analysis
Source: Front Med (Lausanne). 2025 Sep 24;12:1573573. doi: 10.3389/fmed.2025.1573573 (PMC12504262; doi:10.3389/fmed.2025.1573573)
Supplement: Supplementary file 1 [file Data_Sheet_1.PDF]

Table S1

Literature search strategy for the databases searched

Database	Search Strategy
Pubmed	(((("Pregnant Women"[Mesh]) OR (((Pregnant Woman[Title/Abstract]) OR (Woman, Pregnant[Title/Abstract])) OR (Women, Pregnant[Title/Abstract]))) OR (("Pregnancy"[Mesh]) OR ((Pregnancies[Title/Abstract]) OR (Gestation[Title/Abstract])))) AND ((((((((((((((((((Factor, Risk[Title/Abstract]) OR (Risk Factor[Title/Abstract])) OR (Social Risk Factors[Title/Abstract])) OR (Factor, Social Risk[Title/Abstract])) OR (Factors, Social Risk[Title/Abstract])) OR (Risk Factor, Social[Title/Abstract])) OR (Risk Factors, Social[Title/Abstract])) OR (Social Risk Factor[Title/Abstract])) OR (Health Correlates[Title/Abstract])) OR (Correlates, Health[Title/Abstract])) OR (Population at Risk[Title/Abstract])) OR (Populations at Risk[Title/Abstract])) OR (Risk Scores[Title/Abstract])) OR (Risk Score[Title/Abstract])) OR (Score, Risk[Title/Abstract])) OR (Risk Factor Scores[Title/Abstract])) OR (Risk Factor Score[Title/Abstract])) OR (Score, Risk Factor[Title/Abstract])) OR ("Risk Factors"[Mesh]))) AND (("Lupus Erythematosus, Systemic"[Mesh]) AND (((((Systemic Lupus Erythematosus[Title/Abstract]) OR (Lupus Erythematosus Disseminatus[Title/Abstract])) OR (Libman-Sacks Disease[Title/Abstract])) OR (Disease, Libman-Sacks[Title/Abstract])) OR (Libman Sacks Disease[Title/Abstract])))
Embase	('risk factor'/exp OR 'risk factor' OR 'risk factors':ti,ab,kw OR 'relative risk':ti,ab,kw) AND ('pregnant women'/exp OR 'pregnant women' OR 'pregnant woman':ti,ab,kw OR 'pregnancy' OR 'pregnancy' OR 'child bearing':ti,ab,kw OR 'childbearing':ti,ab,kw OR 'gestation':ti,ab,kw OR 'gravidity':ti,ab,kw OR 'intrauterine pregnancy':ti,ab,kw OR 'labor presentation':ti,ab,kw OR 'labour presentation':ti,ab,kw OR 'pregnancy maintenance':ti,ab,kw OR 'pregnancy trimesters':ti,ab,kw OR 'pregnancy':ti,ab,kw) AND ('dermatovisceritism, malignant'/exp OR 'dermatovisceritism, malignant' OR 'disseminated lupus':ti,ab,kw OR 'disseminated lupus erythematodes':ti,ab,kw OR 'disseminated lupus erythematosis':ti,ab,kw OR 'disseminated lupus erythematosus':ti,ab,kw OR 'erythematodes visceralis':ti,ab,kw OR 'lupovisceritis':ti,ab,kw OR 'lupus erythematodes disseminatus':ti,ab,kw OR 'lupus erythematosus disseminatus':ti,ab,kw OR 'lupus erythematosus visceralis':ti,ab,kw OR 'lupus erythematosus, systemic':ti,ab,kw OR 'osler libman sacks disease':ti,ab,kw OR 's.l.e.':ti,ab,kw OR 'sle':ti,ab,kw OR 'systemic le':ti,ab,kw OR 'systemic lupus':ti,ab,kw OR 'systemic lupus erythematodes':ti,ab,kw OR 'systemic lupus erythematosis':ti,ab,kw OR 'systemic lupus erythematous':ti,ab,kw OR 'systemic lupus erythematosus':ti,ab,kw)
Cochrane	([Lupus Erythematosus, Systemic] explode all trees OR Lupus Erythematosus Disseminatus):ti,ab,kw OR (Systemic Lupus Erythematosus):ti,ab,kw OR (Disease, Libman-Sacks):ti,ab,kw OR (Libman Sacks Disease):ti,ab,kw OR (Libman-Sacks Disease):ti,ab,kw AND (Risk Factors] explode all trees OR (Factor, Risk):ti,ab,kw OR (Risk Factor):ti,ab,kw OR (Correlates, Health):ti,ab,kw OR (Health Correlates):ti,ab,kw OR (Populations at Risk):ti,ab,kw OR (Population at Risk):ti,ab,kw OR (Risk Factors, Social):ti,ab,kw OR (Social Risk Factors):ti,ab,kw OR (Risk Factor, Socia):ti,ab,kw OR (Factor, Social Risk):ti,ab,kw OR (Factors, Social Risk):ti,ab,kw OR (Social Risk Factor):ti,ab,kw OR (Risk Factor Scores):ti,ab,kw OR (Score, Risk Factor):ti,ab,kw OR (Risk Scores):ti,ab,kw OR(Risk Score):ti,ab,kw OR (Risk Factor Score):ti,ab,kw OR (Score, Risk):ti,ab,kw) AND ([Pregnancy] explode all trees OR (Pregnancies):ti,ab,kw OR (Gestation):ti,ab,kw) AND ([Pregnant Women] explode all trees OR (Pregnant Woman):ti,ab,kw OR (Woman, Pregnant):ti,ab,kw OR (Women, Pregnant):ti,ab,kw

(continued on next page)

Table S1 *(continued)*

Database	Search Strategy
Web of Science	(Lupus Erythematosus Disseminatus OR Systemic Lupus Erythematosus OR Libman Sacks Disease OR Libman-Sacks Disease) AND (Risk Factors OR Risk Factor OR Social Risk Factors OR Social Risk Factor OR Risk Factor Scores OR Risk Scores OR Risk Score OR Risk Factor Score OR relative risk) AND (Pregnant women OR Pregnant Woman OR pregnancy OR pregnancies OR Gestation)

Table S2

List of Abbreviations

Abbreviation	Full term
aCL	Anti-cardiolipid antibody
Anti-dsDNA	Anti-double stranded deoxyribonucleic acid
aPL	Antiphospholipid antibody
APOs	Adverse pregnancy outcomes
APS	Antiphospholipid syndrome
BA	Birth asphyxia
C3	Complement 3
CHB	Congenital heart block
CI	Confidence interval
EFE	Endocardial fibrotic torsion
FGR	Fetal growth restriction
GH	Gestational hypertension
HCQ	Hydroxychloroquine
HDL	High-density lipoprotein
HDL-P	High-density lipoprotein particle
IUGR	Intrauterine growth restriction
LBW	Low birth weight
LDL	Low-density lipoprotein
LN	Lupus nephritis
NL	Neonatal lupus
NSAID	Nonsteroidal anti-inflammatory drug
NTE	Neonatal lupus syndrome
OR	Odds ratio
PE	Preeclampsia
PIGF	Placental growth factor
PL	Pregnancy loss
PTB	Preterm birth
TG	Triglyceride
SA	Spontaneous abortion
sFlt-1	Soluble fms-like tyrosine kinase-1
SGA	Small for gestational age
SLE	Systemic lupus erythematosus
SLEDAI	Systemic Lupus Erythematosus Disease Activity Index
SS	Sjogren's syndrome
VLDL-P	Very low-density lipoprotein particle

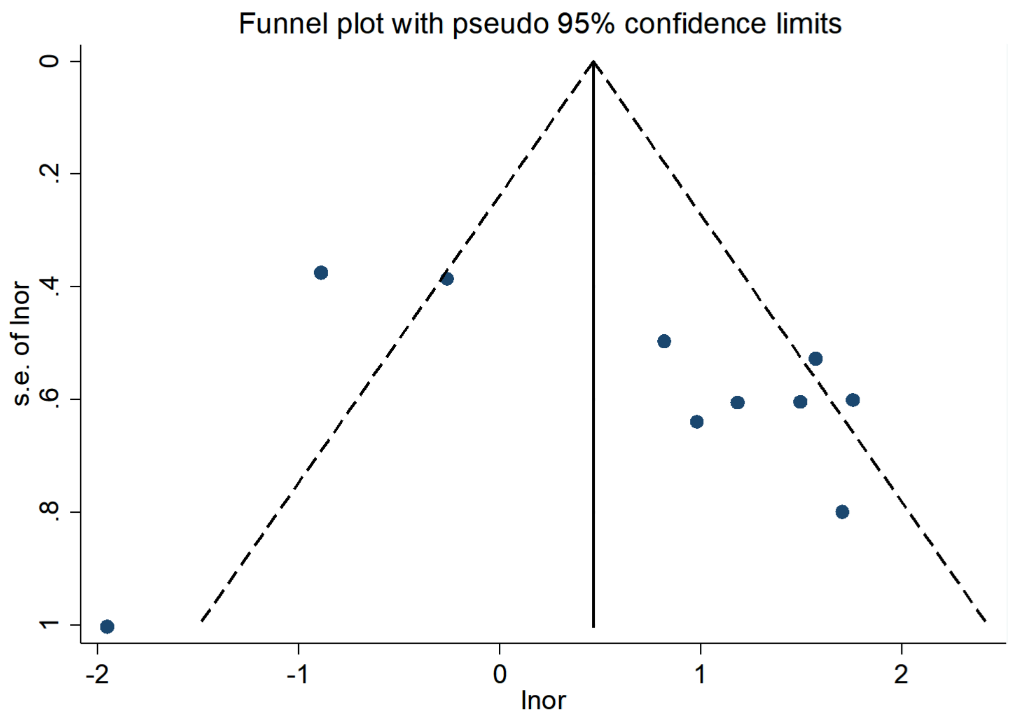

Figure S1: Funnel plot for composite APOs.

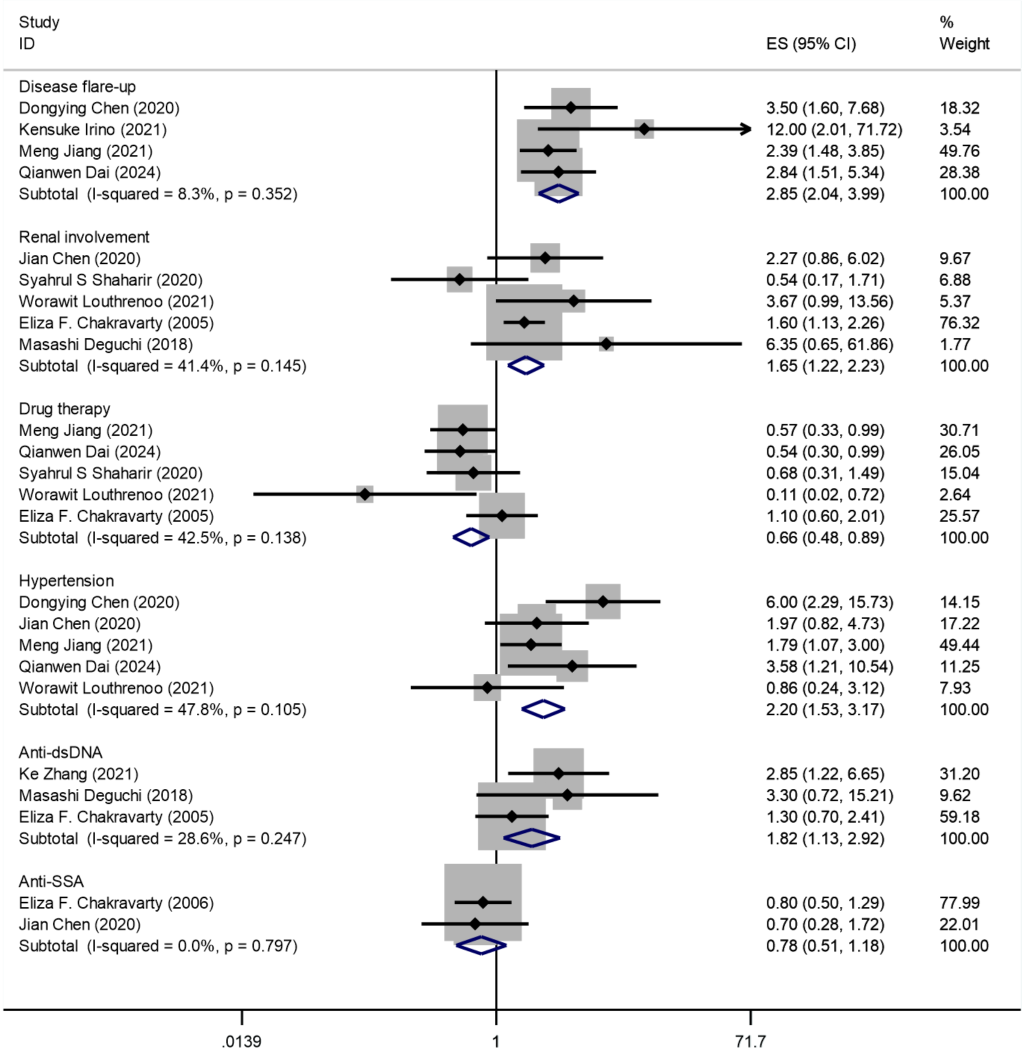

Figure S2: Forest plot presents the association between factors and PTB: odds ratios (ORs) with 95% confidence intervals (CIs).

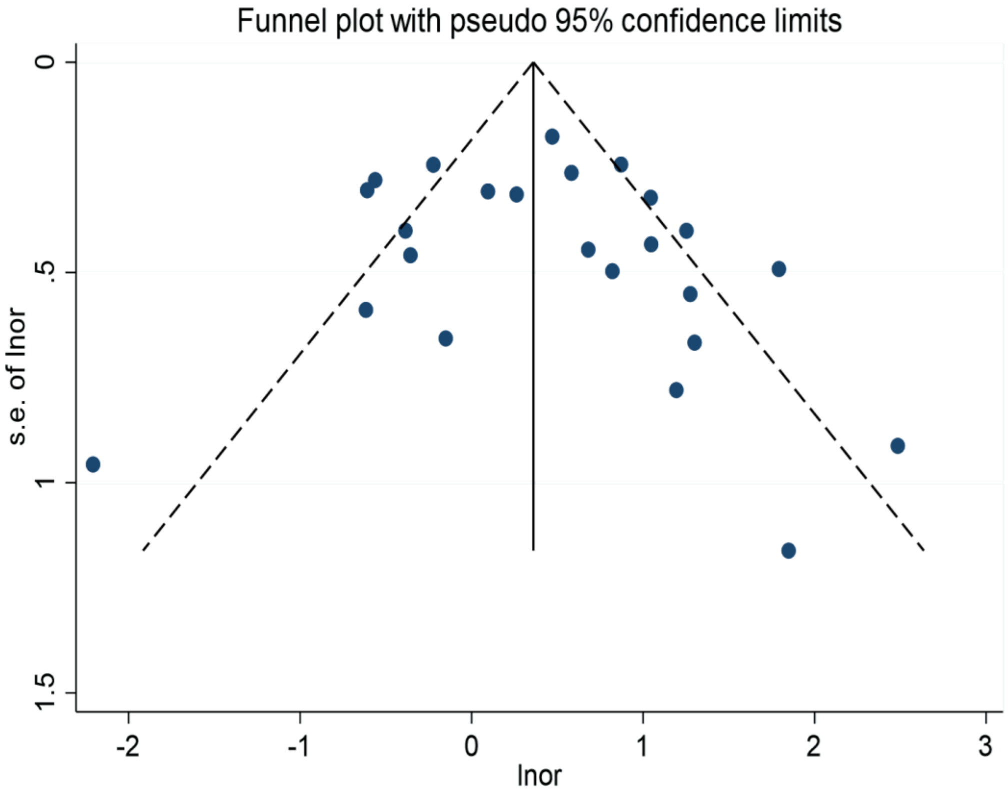

Figure S3: Funnel plot for PTB.

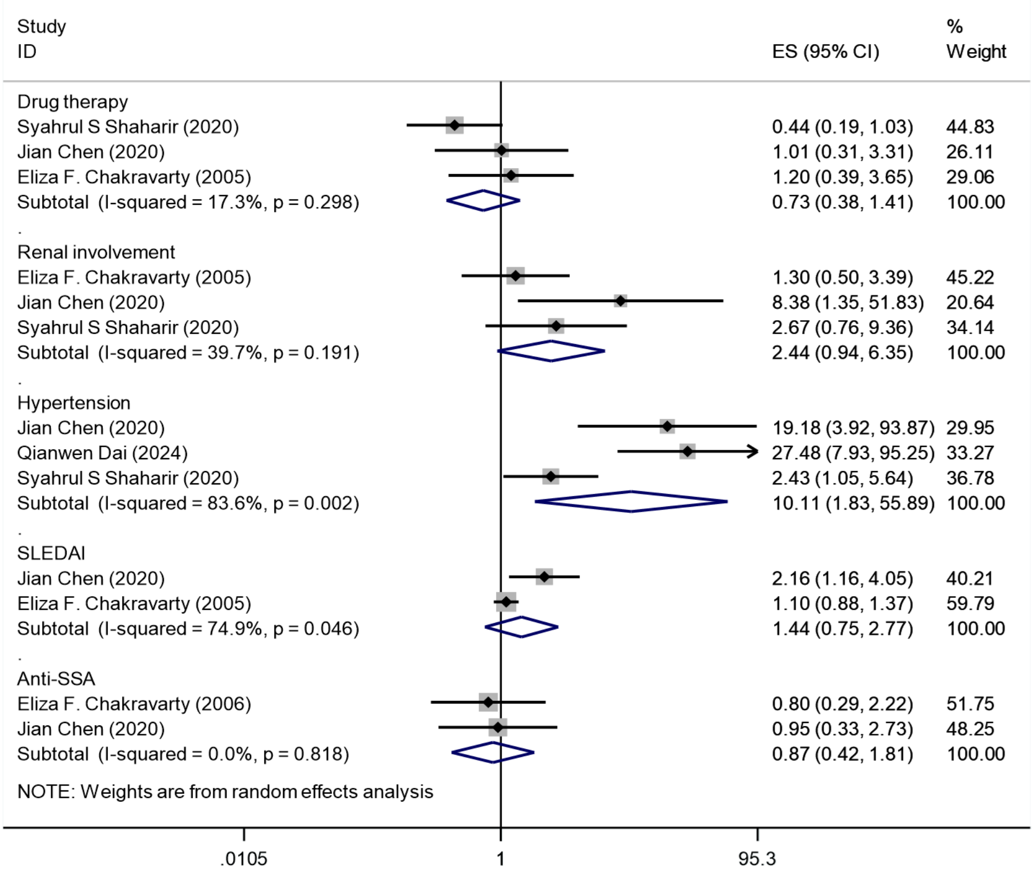

Figure S4: Forest plot presents the association between factors and PE: odds ratios (ORs) with 95% confidence intervals (CIs).

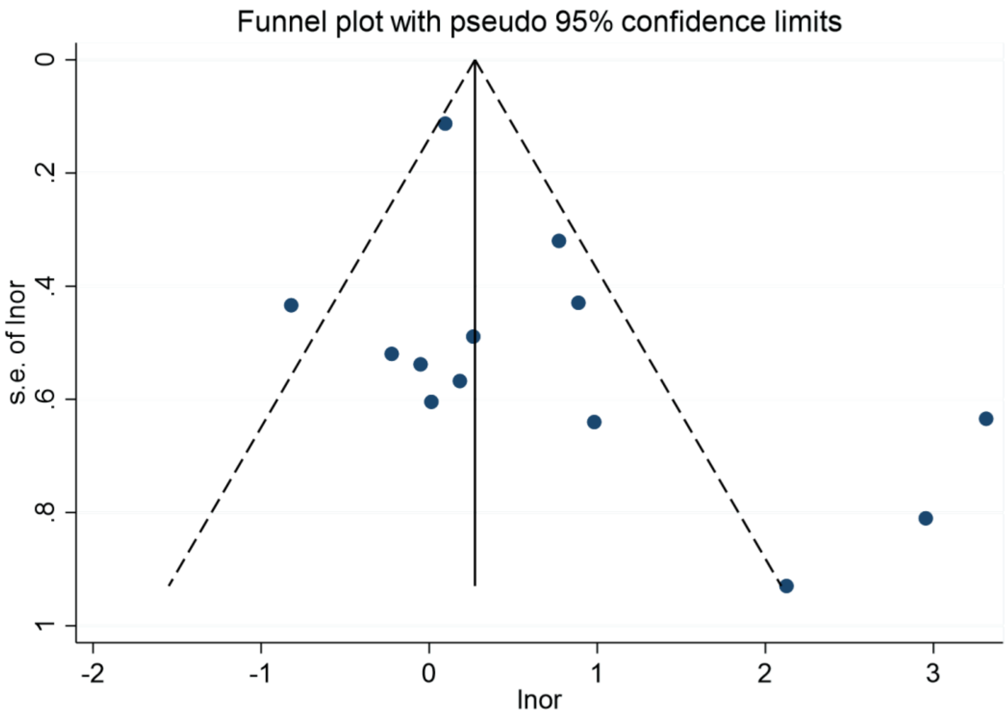

Figure S5: Funnel plot for PE.

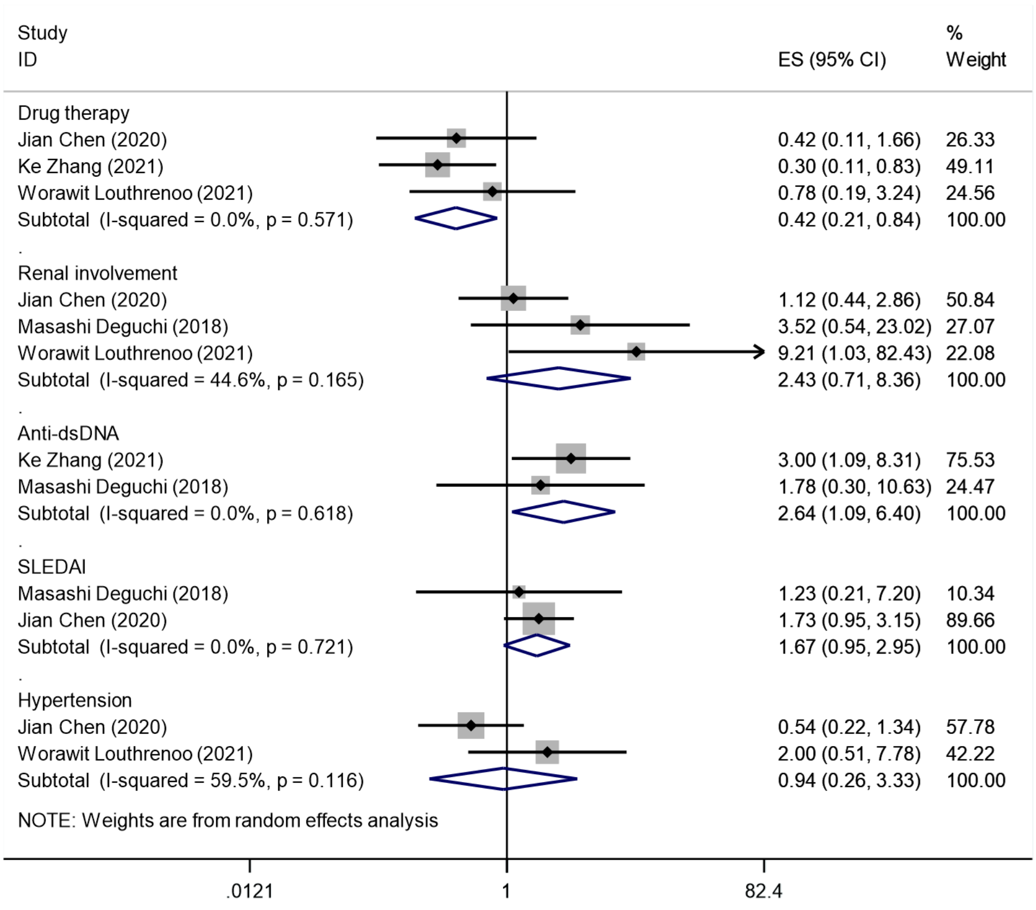

Figure S6: Forest plot presents the association between factors and PL: odds ratios (ORs) with 95% confidence intervals (CIs).

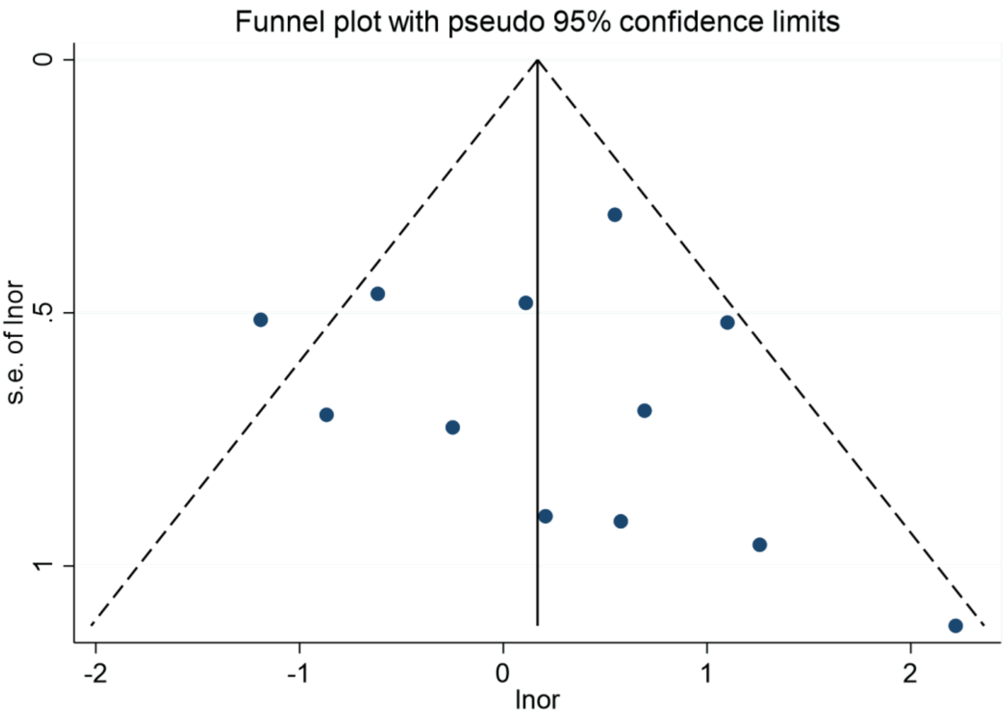

Figure S7: Funnel plot for PL.
